# A Community-Based Cross-Sectional Study on the Epidemiology of Injuries in Raipur City, Chhattisgarh

**DOI:** 10.7759/cureus.41868

**Published:** 2023-07-14

**Authors:** Vipin K Lahare, Nirmal Verma, Aditi Chandrakar, Neha Shrivastava, Monika Dengani, Shubhra A Gupta

**Affiliations:** 1 Community Medicine, Pt Jawaharlal Nehru Memorial Medical College, Raipur, IND; 2 Community and Family Medicine, All India Institute of Medical Sciences, Raipur, IND; 3 Community Medicine, Government Medical College, Mahasamund, IND

**Keywords:** health-seeking behavior, cost, mechanism of injury, nature of injury, injury

## Abstract

Background: Globally, injuries are a major public health concern. An injury is a physical damage that results when the human body is suddenly or briefly subjected to intolerable levels of energy.

Objectives: The objectives of this study are to describe the nature and mechanism of injuries and their association with age and gender and to assess the health-seeking behavior and cost incurred due to mortality and morbidity related to injuries.

Methods and materials: A cross-sectional study focused on the community was conducted in 10 chosen wards of Raipur City. The sample size was 310 injured individuals. The recall period was for a full year. Information was gathered by using a questionnaire that had been pretested. The results were given as percentages, and the association was determined using the chi-square test and Fischer's exact test.

Results: The majority (30.1%) of the study subjects suffered from cut/bite/open wound injuries, followed by fractures (17.3%). The leading type of injury was caused by falls (38.8%) and road traffic injuries (34.9%), followed by burns (7.1%) and dog bites (5.4%). Ninety percent of the study subjects had taken medical care. Half of them (51.3%) visited a private hospital, and 23.1% did not visit any hospital for treatment. Fifty percent of the study subjects or their family had expenses less than Indian National Rupee (INR) 500. A significant association was found between age and fracture and the sprain type of injury. The burn type of injury was more among females, which is significantly associated. A significant association was found between age and injury caused by a dog bite, fall, and traffic. The association between gender and injury caused by traffic, burn, and fall was significant.

Conclusions: Focusing on reducing injury-related morbidity may be crucial in injury prevention techniques including behavioral changes, health education, and the urgent need for the proper implementation and oversight of a road safety act.

## Introduction

"An injury is caused by an acute exposure to physical agents such as mechanical energy, heat, electricity, chemicals, and ionizing radiation interacting with the body in amounts or at rates that exceed the threshold of human tolerance" [1]. As per the World Health Organization (WHO) report, the most common cause of death for people throughout the world is injury. Thousands of individuals die each year because of traffic accidents, homicides, suicides, and other bodily injuries. Each year, five million individuals worldwide lose their lives to accidental or violence-related injuries, which constitute around 9% of all fatalities. Automobile accidents, homicides, and suicide are three of the leading causes of death for people between the ages of five and 29 [2]. The lives of almost 1.3 million people are ended annually by traffic accidents. There are also 200 to 50 million non-fatal injuries, many of which lead to disabilities [3]. The rising number of people living in overcrowded and unsafe settlements, recent advances in industry and the utilization of vehicles, and the lack of accessible and inexpensive health services in an emergency all contribute to an increased burden on the healthcare of individuals in developing nations [4].

In India, cardiovascular disease is the most common cause of death, and there is an increase in the percentage of deaths due to falls (35.1% more deaths) and road injuries (4.9% more deaths) in the year 2019 when compared to 2009 [5]. A significant sociodemographic, epidemiological, technical, and media transformation is taking place in India, which influences its health scenario. Due to socioeconomic growth and development, India has experienced tremendous urbanization, motorization, industrialization, and migration during the past 20 years. Modern technology and mechanization are changing the way people live and work. In India, injuries are thought to be the cause of 15% of all fatalities and 15% of years lived with a disability (disability-adjusted life years {DALYs}). As a result, 15-20 million people are hospitalized, 1.5 million people are projected to die from injuries, and the nation suffers an economic loss of up to 3% of gross domestic product (GDP) [6].

Injury and traffic violations have been prominent causes of death in Chhattisgarh. In Chhattisgarh, traffic accidents claimed the lives of almost 4600 persons in 2020 [7]. Very little community-based epidemiological research on injuries has been done in India and Chhattisgarh; although there are fewer data available on fatal injuries, it is highly challenging to gather information on non-fatal injuries. The first step in lowering the hazards of injury in a community is education. With this context, the current research was carried out to describe the nature and mechanism of injury and its association with age and gender characteristic and to assess the health-seeking behavior and the cost incurred due to morbidity and mortality related to injuries.

## Materials and methods

A cross-sectional study focused on the community was done in 10 selected wards of Raipur City, Chhattisgarh, from January 2022 to June 2022.

The inclusion criteria were the following: (1) study subjects who have been residing in the same address for the past one or more years and (2) those study subjects among whom injuries occurred within the past year.

The exclusion criteria were the following: (1) selected households that are closed at the time of the door-to-door survey, (2) individuals with mental health conditions who are unable to recall the events of the past year, and (3) noncooperative individuals.

A predesigned, semi-structured questionnaire consisting of two parts, which include the sociodemographic profile of the study subject and information about the nature and mechanism of injuries, was used. The type of healthcare facility used after injury by the study subjects was asked and grouped into government, private, and both private and government facilities. The socioeconomic classification was done by using the updated BG Prasad's classification for the year 2021 [8]. The required data was collected through a door-to-door survey using the interview method.

Permission was taken from the "Institutional Ethics Committee" of the Pt Jawaharlal Nehru Memorial (JNM) Medical College, Raipur City, Chhattisgarh (No/MC/Ethics/2021/169). All the study subjects in the household were briefed about the purpose and objective of the research, and verbal consent was taken from all individuals in the household who agreed to participate prior to the interview. The participants were ensured that their identity and information would be kept confidential during and after the study. For this, all the study subjects were given a unique ID to maintain their confidentiality.

Sample size estimation was done by using the WHO report (2004); from a community-based survey, the overall prevalence of injuries was reported as 41.2 per 1000 population, and using the formula as 4pq/L^2^, where p = expected proportion in population based on previous studies (taken as 41.2), taking precision as 15% of expected proportion and nonresponse rate of 25%, the total sample size comes out as 305, which is rounded to 310. A simple random sampling method was used for the selection of wards. There are 10 zones and 70 wards (each zone consisting of seven wards) in Raipur City, and one ward of each zone was randomly selected by lottery method. From each ward, the total number of injured study subjects was chosen by probability proportional to size (PPS) sampling. The selection of households from each ward was done by random walk technique. In the case of nonresponse or non-consent, an adjacent household was included as a replacement. In each selected household, the interview was conducted with the victim in person, but if the victim is not available, then the interview was conducted with any eligible respondent, preferably the head of the family, and if the head of the family is not available, then any member whose age is more than 18 years and available at the time of the survey was interviewed to control for the limitation in the timing of the survey. In case the victim is a child less than 12 years of age, then consent was taken from the parent, and any one parent was interviewed. Three hundred twelve individuals were interviewed from 400 households to find out 312 injuries as no individual reported more than one event of injury in the last year. During the interview, sociodemographic details and a history of injuries in the last year were collected. Data was checked for its completeness and entered in Microsoft Excel (Microsoft® Corp., Redmond, WA). The result was expressed in frequency (n) and percentage (%). Chi-square test and Fischer's exact test were applied to find out the association. P-value < 0.05 was considered as statistically significant.

## Results

The sociodemographic profile of injured individuals was as follows: the mean age of the study subjects was 30.40 ± 18.09 (year ± SD). The majority (21.5%) of the subjects were in the 21-30 years old age group, followed by 1-10 years old (17.6%). Of the study subjects, 61.86% were males, while the rest (38.14%) were females. Of the study subjects, 56.73% belong to the nuclear family. Twenty-six percent of the study subjects were educated up to primary school, followed by 20.5% up to high school, 14.7% up to middle school, and 11.9% up to higher secondary school; 7.7% graduated, and the remaining 1.3% were educated postgraduate and above. Of the study subjects, 11.5% were illiterate, and almost 2.6% of the study subjects were children below five years who are not admitted to any school. The majority of the study subjects were students (26.92%), followed by daily workers (23.71%), private job workers (21.79%), homemaker/housewives (9.93%), retired (2.24%), and government job workers (1.60%). Nine percent of the study subjects were unemployed, and almost 4.8% of the study subjects were preschool children. Of the study subjects, 93.26% were Hindu by religion. The majority (42%) of the study subjects belonged to the middle class, followed by 26% in the lower-middle class, 23% in the upper-middle class, 5% in the upper class, and 4% in the lower class, as per the updated modified BG Prasad's socioeconomic scale for the year 2021 (Table 1).

**Table 1:** Sociodemographic profile of injured individuals

Variables	Frequency (N = 312)	Percentage (%)
1. Age		
01-10	55	17.63
11-20	49	15.71
21-30	67	21.47
31-40	49	15.71
41-50	44	14.10
>50	48	15.38
2. Gender		
Male	193	61.86
Female	119	38.14
3. Education		
Illiterate	35	11.22
Up to 10th standard	212	67.95
Higher secondary school	37	11.86
Graduation and above	28	8.97
4. Occupation		
Unemployed	28	8.97
Preschool	15	4.80
Student	84	26.92
Homemaker/housewife	31	9.93
Daily worker	74	23.71
Private	68	21.79
Government	5	1.60
Retired	7	2.24
5. Socioeconomic status (as per modified BG Prasad's scale for the year 2021)		
I. Upper (≥7863)	16	5.12
II. Upper middle (3931‑7862)	73	23.39
III. Middle (2359‑3930)	130	41.66
IV. Lower middle (1179‑2358)	81	25.96
V. Lower (<1189)	12	3.84

The nature of injury identifies the primary physical characteristic of injury. It basically describes the damage related to the part of the body. The majority (30.1%) of the study subjects suffered from cut/bite/open wound injuries (Table 2). The mechanism of injury refers to the way damage to the skin, muscle, organ, and bones happens. Among the surveyed population, the most common mechanism of injury was by falls (38.8%) and due to traffic injuries (34.9%) (Table 3).

**Table 2:** Distribution according to the nature of injury (N = 312) *Others include penetrating injury and dislocation

Nature of injury	Frequency (N = 312)	Percentage (%)
Cut, bite, and open wound	94	30.1
Fracture	54	17.3
Bruise	39	12.5
Head injury	32	10.3
Sprain/strain	31	9.9
Burn	27	8.7
Contusion	21	6.7
Organ system injury	12	3.8
Others^*^	2	0.6

**Table 3:** Distribution according to the mechanism of injury (N = 312) *Animal bite includes any animal bite other than dogs **Other types of injury include slipping, sports, thermal, and overuse

Mechanism of injury	Frequency (N = 312)	Percentage (%)
Fall	121	38.8
Traffic injury	109	34.9
Others**	24	7.7
Burn	22	7.1
Dog bite	17	5.4
Assault	07	2.2
Electrocution	06	1.9
Snake/animal bite*	3	1.0
Poisoning	1	0.3
Drowning	1	0.3
Choking/hanging	1	0.3

Cut/bite/open wound (36.5%), bruise and contusion (25%), and head injury (14.4%) were three common types of injury in the ≤20 years old age group. In the 21-40 years old age group, cut/bite/open wound (30.1%), fracture (23.2%), and bruise and contusion (16.3%) were the top three types of injury. In the 41-60 years old age group, cut/bite/open wound (23.7%), fracture (22.3), and bruise and contusion (15.7%) were three common injuries, whereas in more than 60 years old age group, sprain (31.2), cut/bite/open wound and bruise and contusion (18.8% each), and fracture (12.5%) were the prevalent types of injuries (Table 4). The leading type of injury in females was cut, bite, and open wound (26.1%), followed by bruise and contusion (19.3%) and fracture (16.8%), while in males, cut/bite/open wound (32.6%), bruise and contusion (19.2%), and fracture (17.6%) were the three leading types of injury (Table 5).

**Table 4:** Association between the nature of injury and age *P-value < 0.05 is significant ^#^Fischer's exact test

Nature of injury	Age (in years)								P-value
	≤20	%	21-40	%	41-60	%	>60	%	
Bruise and contusion	26	25.0	19	16.3	12	15.7	3	18.8	0.33
Burn	10	9.6	10	8.6	7	9.2	0	0	0.64
Cut, bite, and open wound	38	36.5	35	30.1	18	23.7	3	18.8	0.21
Fracture	8	7.7	27	23.2	17	22.3	2	12.5	0.01*
Head injury	15	14.4	9	7.7	5	6.5	2	12.5	0.25
Organ system injury	3	2.9	6	5.1	4	5.2	1	6.2	0.80^#^
Sprain/strain	4	3.8	10	8.6	13	17.1	5	31.2	0.00*

**Table 5:** Association between the nature of injury and gender (N = 312) *P-value < 0.05 is significant

Nature of injury	Gender		P-value
	Female (N, %)	Male (N, %)	
Bruise and contusion	23 (19.3)	37 (19.2)	0.97
Burn	17 (14.3)	10 (5.2)	0.01*
Cut, bite, and open wound	31 (26.1)	63 (32.6)	0.21
Fracture	20 (16.8)	34 (17.6)	0.85
Head injury	10 (8.4)	22 (11.4)	0.39
Organ system injury	2 (1.7)	12 (6.2)	0.06
Sprain/strain	16 (13.4)	15 (7.8)	0.10

Of the study subjects aged ≤20 years, 48.1% were injured by a fall, followed by traffic injury (24.0%) and dog bite (11.5%). In the 20-40 years old age group, most of them were injured by traffic injury (49%), followed by falls (25.9%). In the case of >40 years old age group, the majority of injuries occurred due to falls, followed by traffic injury (Table 6). Falls (47.9%) were the most common cause of injuries in females, followed by traffic injury (25.2%) and burn (10.9%). Traffic injury (40.9%) was the leading cause among males, followed by falls (33.2%) (Table 7).

**Table 6:** Association between age group and the mechanism of injury (N = 312) *P-value < 0.05 is significant **P-value < 0.01 is highly significant ^#^Fischer's exact test

Mechanism of injury	Age (in years)								P-value
	≤20	%	21-40	%	41-60	%	>60	%	
Assault	2	1.9	3	2.6	1	1.3	1	6.2	0.98^#^
Burn	9	8.7	8	6.9	5	6.6	0	0	0.61
Dog bite	12	11.5	3	2.6	2	2.6	0	0	0.03*^#^
Fall	50	48.1	30	25.9	28	36.8	12	75	0.00**
Others	6	5.8	15	12.9	13	17.1	1	6.2	0.09
Traffic injury	25	24.0	57	49.1	27	35.5	2	12.5	0.00**

**Table 7:** Association between the mechanism of injury and gender (N = 312) *P-value < 0.05 is significant **P-value < 0.01 is highly significant ^#^Fischer's exact test

Mechanism of injury	Gender		P-value
	Female (N, %)	Male (N, %)	
Assault	2 (1.7)	5 (2.6%)	>0.05^#^
Burn	13 (10.9)	9 (4.7%)	0.03*
Dog bite	3 (2.5)	14 (7.3%)	0.07
Fall	57 (47.9)	64 (33.2%)	0.00**
Others	14 (11.8)	22 (11.4%)	0.92
Traffic injury	30 (25.2)	79 (40.9%)	0.00**

Of the study subjects, 40.4% have sustained injury on the highway, followed by 40.1% at their homes and 18.3% in the working place. The rest (1.3%) of the study subjects were injured in school (Table 8). In this study, the majority of the injuries occurred in the daytime or afternoon (27.9%), followed by evening (26.9%) and morning (26.6%), and the rest (18.8%) of the injuries occurred at night.

**Table 8:** Distribution according to the place of injury (N = 312)

Places where the injury occurred	Frequency (N = 312)	Percentage (%)
Home	125	40.1
School	4	1.3
Highway (road)	126	40.4
Working place	57	18.3

**Figure 1:**
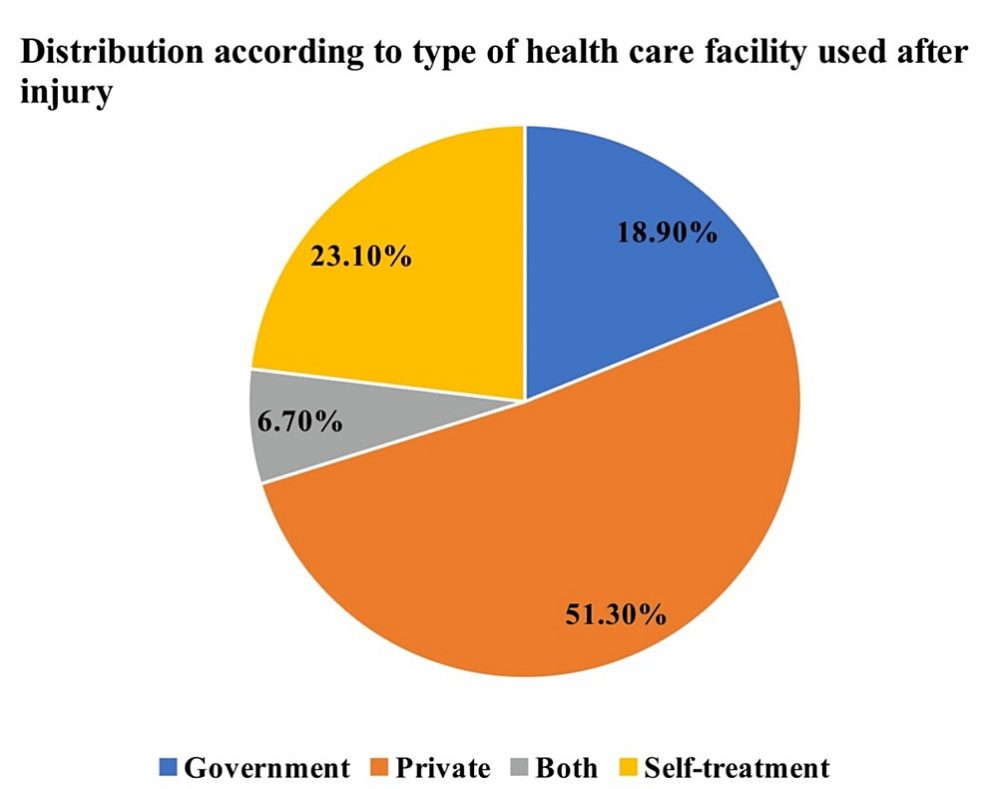
Type of healthcare facility used by the study subjects after injury (N = 312)

**Figure 2:**
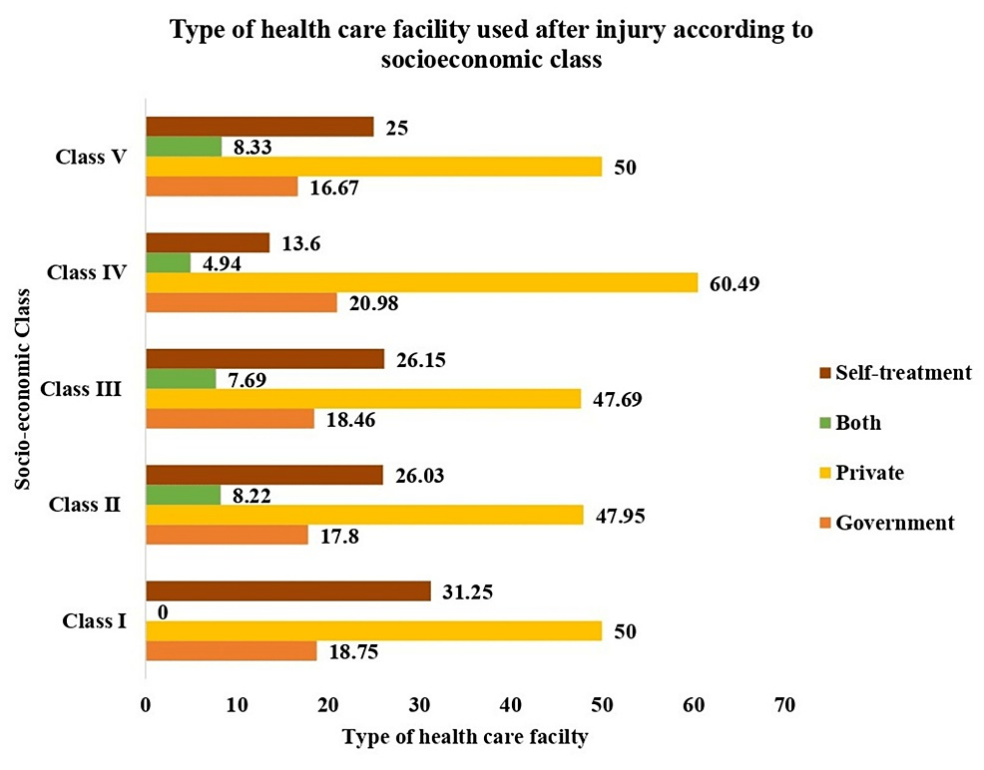
Type of healthcare facility used after injury according to socioeconomic class

The health-seeking behavior of the study subjects revealed that out of 312 study subjects, the majority (90.4%) had taken medical care, and 9.6% of injured persons had not taken any treatment care after injury. Of the study subjects, 51.30% visited private hospitals, and 23.10% did not visit any hospital (self-treatment) for treatment. Only 18.90% used government hospitals for treatment, and 6.70% of the study subjects visited both private and government health facilities (Figure 1). The majority of them used private facility after injury irrespective of their socioeconomic class (Figure 2). Fifty percent of the study subjects or their family had expenses less than Indian National Rupee (INR) 500 or US dollar (USD) 6.10; 17.6% of the study subjects incurred treatment costs between INR 500 (USD 6.10) and INR 1000 (USD 12.20) and 17.3% between INR 1000 (USD 12.20) and INR 10000 (USD 122.03). The rest (14.7%) of the study subjects incurred more than INR 10000 (USD 122.03).

## Discussion

Research in injury has received little attention in developing nations. The present research was done to illustrate the epidemiological factor, nature, and pattern of injuries; health-seeking behavior; and cost incurred after injury in the urban community of Raipur City, Chhattisgarh. In the present study, 30% suffered from cut/bite/open wound injuries, followed by fracture (17.3%), bruise (12.5%), head injury (10.3%), sprain/strain (9.9%), and burn (8.7%). Of the study subjects, 6.8% have not specified which type of injury they suffered. A study done by Rao and Mahajan [9] also found similar types of results in which 19.9% of the study subjects had cut injuries, followed by fractures in 18.9%; laceration and abrasions in 15.3% and 13.8%, respectively; burn injuries in 8.3%; contusion in 9.7%; and penetrating injury in 7.9%. Six percent had concealed injuries, and the rest of the injury subjects (3.6%) had another type of injury such as blunt trauma.

In the present analysis, the most common mechanism by which injury occurred was by fall at 38.8%, while in 34.9%, it was due to traffic. Traffic injuries are common in the study area as Raipur City is developing and a capital city of a state. This may be partly due to the increase in the number of vehicles on the road, which leads to a high chance of collision due to less spacing; other factors may be due to the passing of national highways through the city with unsafe service roads, no speed limit signage, the interaction of bridge without a sign, and sometimes traffic signals not working properly. Traffic injury is the most common type of injury in Raipur City, which can be prevented by the strict enforcement of traffic laws. Of the study participants' injuries, 7.1% were caused by burn, 5.4% by a dog bite, and 2.2 by assault. A similar study done by Blankson et al. showed that road traffic accidents (39.1% of the injuries) and falls (19.7%) were each responsible for the majority of the injuries. followed by violence/assault (12.0%), burn (3.9%), and animal bite (1.8%) [10]. Another study done by Chowdhury et al. showed that the major mechanism of injury in 30% of the subjects was road accidents, followed by slip/trip/fall in 29.5% and a collision with a person or object in 17.9%. A deep cut occurred in 10% of the cases, burns in 3.3%, electrocution in 1.7%, drowning in 0.7%, animal attack in 0.9%, and bites or stings from insects in 1.2% [11].

The present study shows that cut/bite/open wound was the most common type of injury in the age group of 0-60 years, while in the age group of >60 years, sprain was the prevalent type of injury. Sprain is more common in the older population due to tripping, gait, and balance problems. A study done by Kobusingye et al. found that burns and falls were the leading types of injuries in children of ages 10 years or younger (41%), while abrasion and fracture-type injuries were the leading types among 30-39 years old [12]. As their age increases, they devote themselves to different occupations, which leads to more exposure to the outer environment.

This study shows that 41.8% of study subjects of ages ≤20 years were injured by falls, followed by traffic injury (24%). This may be due to more involvement in outdoor activities such as sports and play. Children are more prone to fall because of their impulsiveness, their lack of experience in the calculation of risk, and their natural curiosity. In the age group of 20-40 years, most of them were injured due to traffic injury. Risk-taking is very normal in the young age group. Traffic injuries most commonly seem to be due to two factors: one due to traffic environment such as unsafe road infrastructure and increase in the number of vehicles on the road and the other due to human factors such as overspeeding, the nonuse of motorcycle helmets and seat belts, distracted driving such as the use of mobile phones, and driving under the influence of alcohol and other psychoactive substances. For those who are >40 years old, most of them are injured by falls, followed by traffic injuries. People are more prone to fall with increase in age, as eyesight, hearing, and reflexes are not very sharp as they were in the younger age group, and also, there is an increased problem of gait and balances with increase in age. A study done by Kobusingye et al. [12] found that falls were the leading cause of severe injuries in children aged 10 and under, accounting for 33% of all severe injuries, followed by traffic (27%) and burns (13%), and traffic was the leading cause in the age group 20 and older, while those aged 30-39 were the most affected by traffic, followed by those aged 20-29 and 40-49 (29% each). A study done by Mahalakshmy et al. [13] reported that the most common injuries that occurred among those <18 years old were falls (7.2%), bites by scorpions/snakes/dogs (5.1%), and burns (2.8%). In the age group of 18-60 years, common injuries were bites by scorpions/snakes/dogs (8.4%), falls (7.1%), and road traffic accidents (7.0%). However, among the older population, those more than 60 years old were injured by falls (9.8%), and 9% had bites by scorpions/snakes/dogs. A study done by Olawale and Owoaje found that traffic injuries were the leading source of injury across all age groups, with the exception of children aged 5-14, where falls were the leading cause of injury [14].

In this study, the most common cause of injury among females was due to falls, while in the case of males, the leading cause was traffic injury, followed by falls. This may be due to the more involvement of males in outdoor activities due to their occupation than females. Similar results were found in the study conducted in Pondicherry by Mahalakshmy et al. [13]. Males had a high incidence of road traffic accidents (9.1%) and falls (8.1%), whereas females had a high prevalence of insect/scorpion bites (8.1%) and falls (6.7%). A study done by Olawale and Owoaje [14] reported that falls and traffic injuries (both 25%) were the major causes of injury among males, followed by blunt injuries (15%) and cuts/stabs (21%). Cuts/stabs (24%) were the most common causes of injury among females, followed by road accidents (22%). A study conducted by Bangdiwala et al. noted that falls are a significant cause of injuries among boys and young males in developing nations, and the male-to-female ratio was 2:1 [15]. Another study by Bolandparvaz et al. reported that vehicle accidents, motorcycle accidents, and falls were the most prevalent injury mechanisms in the male group, while car accidents, falls, and pedestrian accidents were the most common in the female group [16].

In this study, most of the injuries are sustained at highways and homes. The possible reason may due to the overspeeding of vehicles and more number of intersection on highways due to the passing of national highways through the city. A study done by Sharma et al. found that the majority of injuries occurred at home (32.26%), followed by road or highway injuries (29.3%) and industrial area injuries (12.90%) [4]. Another study by Cardona et al. revealed that the home accounted for 38.6% of injuries, followed by the road (30.5%), the workplace (27.4%), and other locations (3.5%) [17]. In this study, injuries are more common in daytime, while Chowdhury et al.'s research has shown that daytime hours are when most injuries take place. The peak injury period was from 10 am to 6 pm (76.7%) and then from 7 pm to 8 pm (7.6%) [11].

The present study shows that the majority of injured individuals visited private hospitals after injury, but a study done by Mahalakshmy et al. [13] showed that the majority (58.6%) of injured subjects were taken to government facility treatment and 18.8% to private doctors, 15% used home remedies, and 7.7% went to the village-level faith healers, while another study done by Lamawansa and Piyathilake [18] observed that about 49.4% of injured persons took treatment at the government facility and 23.5% took treatment in private institutions. In this study, 50% of the study subjects or their family had expenses less than INR 500/-; among 17.6% of study subjects, the cost of treatment was between INR 500 and 1000/- and 17.3% between INR 1000/- and 10000/-. The rest (14.7%) of the study subjects incurred more than INR 10000/-. A similar study done by Rao and Mahajan [9] showed that 32.4% of the study subjects' cost of treatment of injury was less than INR 500/-, 10.8% of the study subjects' cost of treatment of injury was between INR 500 and 1000/-, and 51.4% of study subjects' cost of treatment of injury was between INR 1000 and 10000/-. A study done by Gumber [19] showed that the average cost of hospital-based treatment in the private sector was 2.5 times higher than in the public sector.

The result of the present study should however be viewed with the limitation of long recall period. Recall bias is very likely, especially in the cases of less severe injuries. Injury incidents are frequently underreported when proxy respondents are used in the place of first-choice respondents. Additionally, memory deterioration could be more severe if a respondent is speaking for multiple people.

## Conclusions

This study was done to compile injury epidemiology data. Injury affects people of all ages, although it most frequently affects those between the ages of 21 and 30. The most common types of injuries in Raipur City, Chhattisgarh, that cause morbidity were falls and traffic accidents. Health awareness regarding injury and accident prevention should be done as there is enough scientific evidence available for what can be done to stop them from happening. The present cross-sectional study was done in the urban area of Raipur City; a longitudinal study can be planned to know the causal risk factors of different types of injuries.
